# The prognostic value of PCNA expression in patients with osteosarcoma: A meta-analysis of 16 studies

**DOI:** 10.1097/MD.0000000000008254

**Published:** 2017-10-13

**Authors:** Xing Wang, Dong Wang, Na Yuan, Fanxiao Liu, Fu Wang, Bomin Wang, Dongsheng Zhou

**Affiliations:** a Department of Orthopedics, Shandong Provincial Hospital Affiliated to Shandong University, Jinan; b Department of Orthopedic Surgery, Laiwu Hospital Affiliated to Taishan Medical College, Laiwu; c Department of Orthopedics, Zoucheng People's Hospital, Zoucheng; d Department of Orthopedics, Yankuang Group General Hospital, Zoucheng, Shandong, China; e Department of Orthopedic Surgery, Physical Medicine and Rehabilitation, University of Munich (LMU), Munich, Germany.

**Keywords:** disease-free survival, meta-analysis, osteosarcoma, overall survival, proliferating cell nuclear antigen, prognosis

## Abstract

**Background:**

Numerous studies have attempted to determine the prognostic role of proliferating cell nuclear antigen (PCNA) expression in patients with osteosarcoma with no consistent conclusion. We performed this meta-analysis to systematically elucidate the association in a more precise manner.

The purpose of this meta-analysis is to determine the prognostic role of PCNA in patients with osteosarcoma.

**Methods:**

A systematic search of relevant studies was performed in 6 electronic databases including PubMed, Embase, Web of Science, Wanfang database, China National Knowledge Internet (CNKI) database, and Chinese Biological Medical (CBM) Database (up to March 1, 2016) with the following keywords: (PCNA OR proliferating cell nuclear antigen) AND (osteosarcoma OR osteogenic tumor). A manual search of references on relevant articles was also conducted by 2 investigators independently. We performed a comprehensive evaluation of the correlation between PCNA expression and overall survival (OS) or disease-free survival (DFS) by calculating relative ratios (RR) and their corresponding 95% confidence intervals (CI) using STATA software. A fixed- or random-effect model was chosen based on the between-study heterogeneity.

**Results:**

In total, 16 studies with 691 osteosarcoma patients were included in this meta-analysis. PCNA overexpression was found in approximately 57.31% of the patients with osteosarcoma. The meta-analysis suggested that PCNA overexpression in osteosarcoma patients is associated with low OS, but not significantly with DFS (RR = 1.82, 95% CI 1.53–2.18, *P* = .000; RR = 1.15, 95% CI 0.91–1.44, *P* = 0.234). Sensitivity analysis for OS and DFS showed no significant difference and the pooled RRs were stable when the included studies were removed one by one. Similar results were also obtained for subgroup analysis based on different follow-ups and cutoffs to determine PCNA expression.

**Conclusion:**

The findings from this meta-analysis indicate that PCNA overexpression is an effective biomarker for poor prognosis in patients with osteosarcoma for OS. Hence, more large-scale studies are still needed to further warrant this conclusion.

## 1 Introduction

Osteosarcoma is the most common primary malignant bone tumor with higher mortality in teenagers.^[1]^ Treatment of osteosarcoma has been improved greatly with the use of multiple chemotherapeutic agents before definitive resection of the primary tumor.^[2]^ However, approximately 50% of the patients with osteosarcoma face multidrug resistance and poor clinical outcome,^[3]^ with the 5-year overall relapse-free survival rate being 65%.^[4,5]^ Although its occurrence and development are regulated by genetic factors,^[6]^ the prognostic mechanism in osteosarcoma patients remains unclear. Thus, the identification of osteosarcoma prognostic markers and therapeutic targets is an urgent requirement.^[7]^ Recently, several common markers^[8,9]^ have been identified correlated with the metastasis and prognosis in osteosarcoma, and proliferating cell nuclear antigen (PCNA) is a promising marker among these.

Tumor antigens play an important role in tumor occurrence, development, and dispersal process, of which PCNA is the most important. In 1978, Miyachi et al^[10]^ discovered antibodies against PCNA in the sera of patients with Cazenave lupus. PCNA is a cell cycle regulatory protein, and its expression increases significantly in the G1 phase, reaching a peak and decreases in the G2-M phase of cell cycle.^[11]^ PCNA expression showed a periodic change with the replication of DNA phase change, which plays an important role in cellular transition from the G1 phase to S phase. PCNA has high expression in almost all tumor tissues because of its function. Therefore, numerous studies have reported that PCNA expression could accurately reflect the status of cell proliferation, and can be used as a biomarker for the diagnosis and prognosis of malignant tumors.^[12,13]^

Several studies have been performed to explore the association between PCNA expression status and prognosis in osteosarcoma. Some earlier studies showed that high PCNA expression was associated with poor prognosis in the overall survival (OS) of patients with osteosarcoma. However, several other studies suggested that it had no significance. Nevertheless, these results are so contradictory that the significance of overexpression for osteosarcoma prognosis is limited and unconvincing. Therefore, we conducted a meta-analysis to further investigate this prognostic value, and discussed the possibility of PCNA as a prognostic medical marker in osteosarcoma.

## 2 Materials and methods

### 2.1 Data sources and search strategy

In total, 6 electronic databases including PubMed, Embase, Web of Science, Wanfang database, CBM (Chinese Biomedical Literature Database), and CNKI (China National Knowledge Infrastructure) were searched for all potential relevant articles published before March 1, 2016 without any language restrictions. The following search terms were adopted in the meta-analysis: (PCNA OR proliferating cell nuclear antigen) AND (osteosarcoma OR osteogenic tumor). A manual information retrieval was also conducted by 2 investigators (FL and XW) to recognize references of all potentially eligible studies.

### 2.2 Inclusion criteria

Studies were included if they fulfilled the following criteria: patients with osteosarcoma, providing enough data to calculate relative ratios (RRs) and their 95% confidence intervals (CIs), estimating the correlation between PCNA expression and OS or DFS, and case reports, systematic reviews, and letters were all excluded. All disagreements were resolved though discussion and consensus by 2 investigators (FL and XW). Only the most recent or complete study was enrolled if certain article was published duplicate.

### 2.3 Endpoints of interest

The OS and DFS of patients with osteosarcoma were the primary endpoints. No available data for DFS in other years were collected. Subgroup analyses were classified by PCNA expression status using cutoffs defined by respective studies.

### 2.4 Data extraction

All data were extracted from the 16 included studies, and were entered to a predesigned and standardized excel file independently by 2 investigators (FL and XW). These valuable details included the first author's family names in the original articles, year of publication, number of patients, gender, age, inclusion period, Enneking stage, method of evaluating PCNA, PCNA cutoff, follow-up, RR with 95% CI for OS and DFS at different years in osteosarcoma. Only the most recent or complete study was enrolled if a certain article was published in duplicate. All disagreements were resolved through discussion and consensus by 2 investigators (FL and XW).

### 2.5 Quality assessment

The methodological qualities of eligible studies were estimated using Newcastle-Ottawa Scale (NOS)^[14]^ which considered 9 factors. A score of 1 was given to a study for each item. The quality scale ranged from a score of 0 to 9 and studies with high scores were considered as good reports. Studies with scores ≥7 were regarded as high-quality reports and the others were considered as low-quality reports.

### 2.6 Statistical analysis

The correlation between PCNA expression and prognosis of patients with osteosarcoma was evaluated by calculating the pooled RR and its 95% CI. The between-study heterogeneity of our meta-analysis was assessed using the *I*^2^ statistic, a quantitative measure describing inconsistency across studies from 0% to 100%.^[15]^ When heterogeneity was significant (*I*^2^ > 50%), the potential sources of heterogeneity were identified by analyzing the methodological variability of the included studies or by omitting studies one by one to evaluate the impact of a single trial on the overall pooled estimate. If the heterogeneity across studies still existed, a random-effect model was used to calculate the pooled RR and its 95% CI. When the heterogeneity was low (*I*^2^ < 50%), a fixed-effect model was applied. Egger test was used to evaluate the possibility of publication bias. The software STATA, version 12.0 (StataCorp, CollegeStation, TX), was applied for statistical analysis, and *P* <.05 was regarded as not statistically significant.

### 2.7 Ethical statement

Our meta-analysis conformed to the Preferred Reporting Items for Systematic Reviews and Meta-Analyses (PRISMA) statement.^[16]^ Ethical approval or patient consent is not required for conducting this meta-analysis.

## 3 Results

### 3.1 Search results and study characteristics

**Figure 1:**
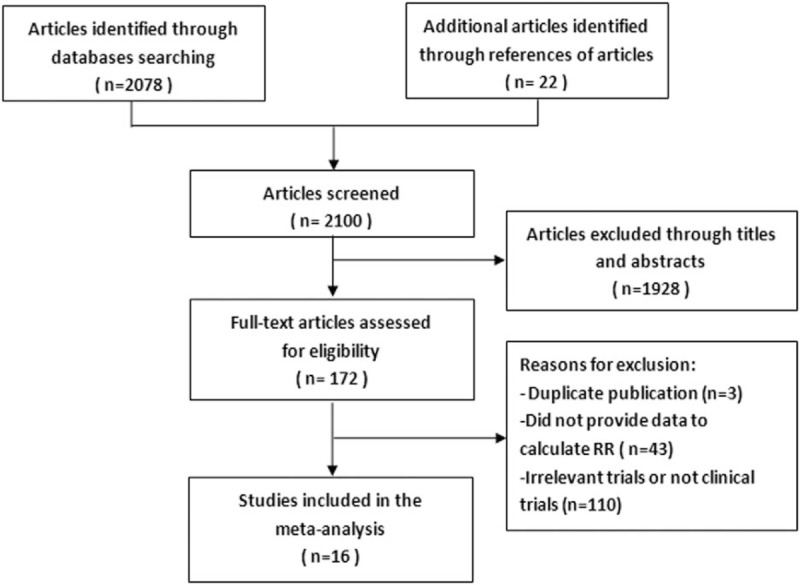
Selection process of eligible studies in the meta-analysis.

In total, 2100 relevant articles were collected by screening the titles and abstracts, of which 2084 were excluded for duplicate publication and various reasons (reviews, letter, congress, case report, or irrelevant to this analysis). After all the full-texts of potentially relevant studies were downloaded, 43 articles were removed because they did not provide enough data to calculate the RRs and their 95% CIs. Finally, 16 studies^[17–32]^ published between 1999 and 2011 with 691 patients (ranging 20–71 in each study, median 41.5) were selected for the meta-analysis. The detailed selection process for the included studies is presented as a flow diagram in Figure 1.

Among these, 5 studies were published in English^[17–21]^ and 11 were published in Chinese.^[22–32]^ The result of PCNA overexpression was found in approximately 57.31% of the osteosarcoma patients. Thirteen studies^[17,18,21–30,32]^ reported the data of OS and 6 studies provided^[17,19–21,29,31]^ the DFS of patients with osteosarcoma. Based on the NOS score, 5 studies got a score of 8, 8 studies achieved a score of 7, and 3 studies had a score of 6. The mean NOS score for these studies was 7.125 (range 6–8). The main characteristics of the 16 included studies are presented in Table 1.

**Table 1:**
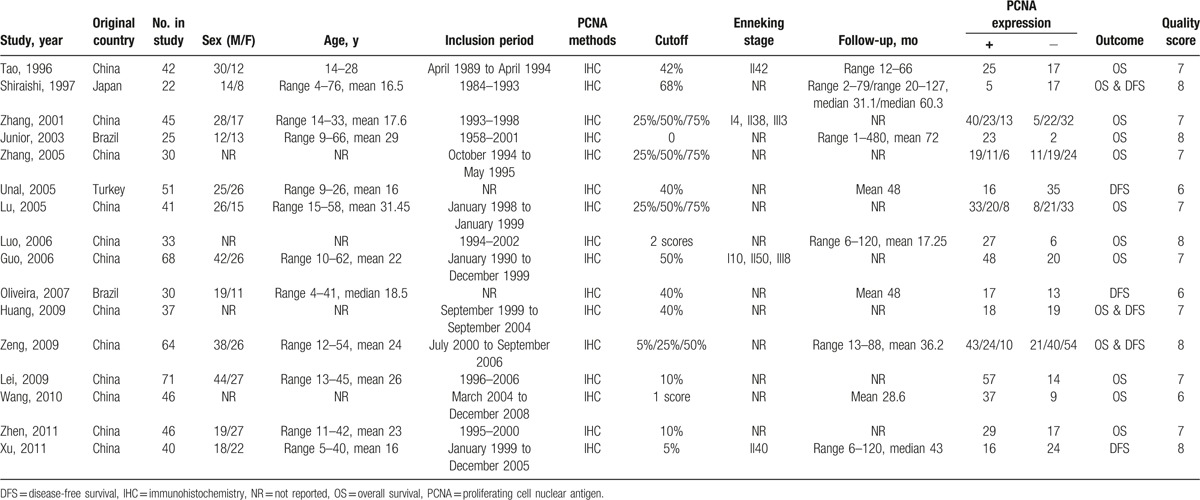
Characteristics of enrolled studies in the meta-analysis.

### 3.2 Correlation between PCNA expression and OS

**Figure 2:**
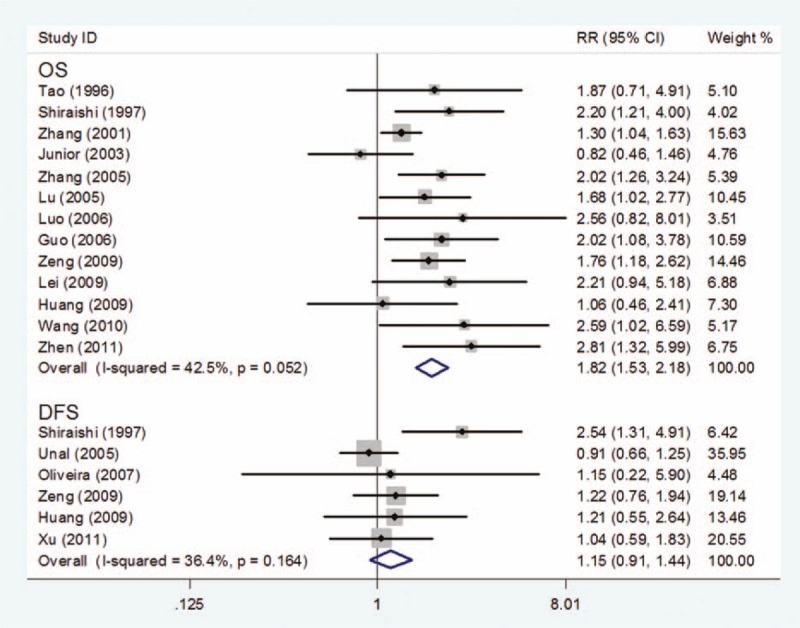
Forest plot of the association of PCNA expression and OS or DFS in osteosarcoma. DFS = disease-free survival, PCNA = proliferating cell nuclear antigen, OS = overall survival.

In total, 13 studies^[17,18,21–30,32]^ explored the association between PCNA expression and OS with enough accessible data to calculate the RRs and 95% CI. The result showed that patients with high PCNA expression had a lower OS than those with low PCNA expression (RR = 1.82, 95% CI 1.53–2.18, *P* = .000) (Fig. 2). We adopted a fixed-effect model to pool the data and found no obvious between-study heterogeneity (*I*^2^ = 42.5%, *P* for heterogeneity = .052).

### 3.3 Correlation between PCNA expression and DFS

**Figure 3:**
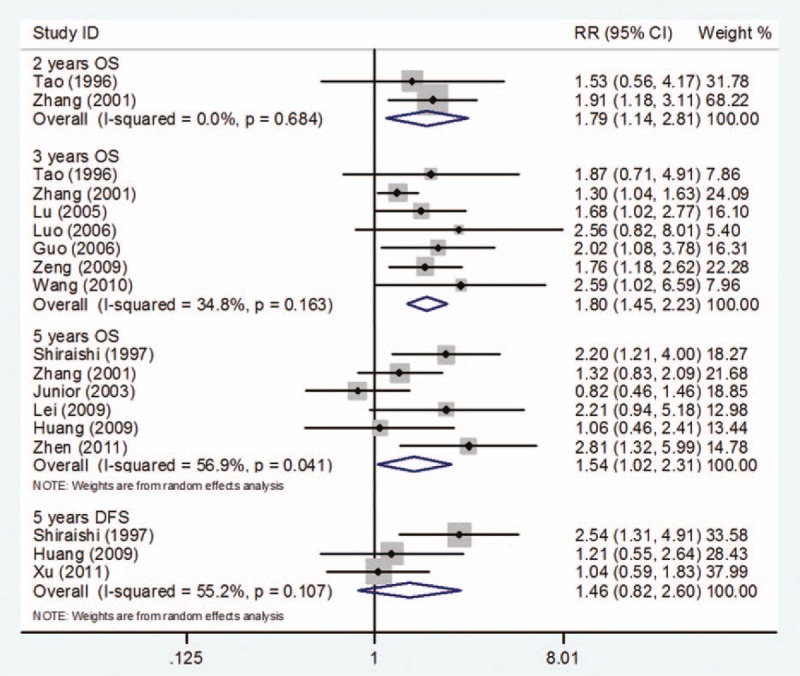
Survival outcome at different years by PCNA expression. PCNA = proliferating cell nuclear antigen.

Six studies^[17,19–21,29,31]^ explored the association between PCNA expression and DFS with enough accessible data to calculate the RRs and 95% CI. Meta-analysis of these 6 studies showed no statistical association between PCNA overexpression and DFS of osteosarcoma (RR = 1.15, 95% CI 0.91–1.44, *P* = .234) (Fig. 3). A fixed-effect model was used for statistical analysis, and no significant heterogeneity was found (*I*^2^ = 36.4%, *P* for heterogeneity = .164).

### 3.4 Subgroup analysis based on different follow-ups

#### 3.4.1 Two-year OS

Only 2 studies^[22,23]^ were searched and included in this subgroup analysis for 2-years OS. Compared with low PCNA expression, high PCNA expression was associated with a poor prognosis of osteosarcoma (RR = 1.79, 95% CI 1.14–2.81, *P* = .011). There was no evidence of statistically significant heterogeneity with a fixed-effect model (*I*^2^ = 0%, *P* for heterogeneity = .684) (Fig. 3).

#### 3.4.2 Three-year OS

In total, 7 studies^[22,23,25–27,29,30]^ provided data for OS at 3 years. Meta-analysis of these 7 studies suggested that PCNA expression was obviously associated with a lower OS rate in patients with osteosarcoma (RR = 1.80, 95% CI 1.45–2.23; *P* = .000). No evidence of statistically significant heterogeneity (*I*^2^ = 34.8%, *P* for heterogeneity = .163) was found between the studies (Fig. 3).

#### 3.4.3 Five-year OS and DFS

Six studies^[17,18,21,23,28,32]^ provided data for OS at 5 years. Osteosarcoma patients with low PCNA expression were found to have higher OS than those with high PCNA expression (RR = 1.54, 95% CI 1.02–2.31; *P* = .038). However, the heterogeneity of between-study was found when a fixed-model was used (*I*^2^ = 56.9%, *P* for heterogeneity = .041), and so, a random-effect model was chosen (Fig. 3).

Only 3 studies^[17,21,31]^ reported DFS information of 5 years. Meta-analysis of these 3 studies showed that PCNA expression status did not influence the 5-year DFS (RR = 1.46, 95% CI 0.82–2.60; *P* = .194). Evidence of between-study heterogeneity was found (*I*^2^ = 55.2%, *P* for heterogeneity = .142) when a fixed-effect model was used (Fig. 3).

### 3.5 Subgroup analysis based on different cutoffs to determine PCNA expression

Differences between subgroups were assessed according to cutoffs for defining PCNA expression.

#### 3.5.1 Cutoff >5% in DFS

**Figure 4:**
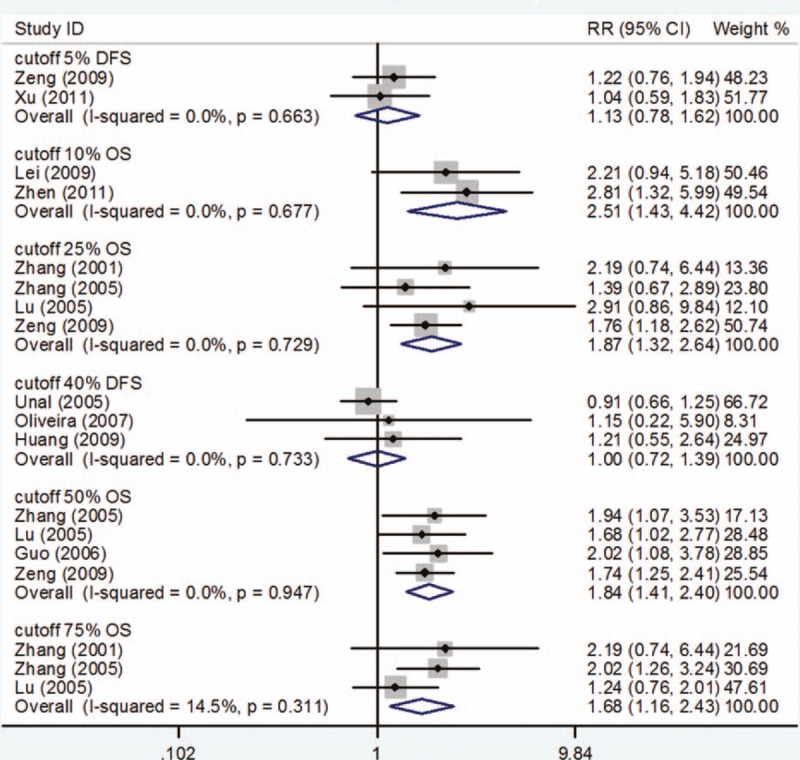
Survival outcome on different cutoffs to determine PCNA positivity. PCNA = proliferating cell nuclear antigen.

Only 2 studies^[29,31]^ provided data for DFS with cutoff > 5% for determining PCNA expression. The results suggested that PCNA expression was not associated with DFS in patients with osteosarcoma (RR = 1.13, 95% CI 0.78–1.62; P = 0.527). No evidence of statistically significant heterogeneity (I-square = 0.0%, P for heterogeneity = 0.663) was found between studies (Fig. 4).

#### 3.5.2 Cutoff >10% in OS

Only 2 studies^[28,32]^ reported data for OS with cutoff *>*10% for determination of PCNA expression. Compared with low PCNA expression, high PCNA expression was associated with poor prognosis for OS (RR = 2.51, 95% CI 1.43–4.42; *P* = .001). *P* for heterogeneity was .677 with *I*^2^ = 0.0% and no between-study heterogeneity was found (Fig. 4).

#### 3.5.3 Cutoff >25% in OS

With a cutoff *>*25% for determining PCNA expression, there were 4 studies^[23–25,29]^ that assessed the association between PCNA and OS. Meta-analysis of these 4 studies showed that high PCNA expression was associated with a statistically significantly poor prognosis for OS (RR = 1.87, 95% CI 1.32–2.64; *P* = .000). No evidence of statistically significant heterogeneity (*I*^2^ = 0.0%, *P* for heterogeneity = .729) was found between studies (Fig. 4).

#### 3.5.4 Cutoff >40% in DFS

Only 3 studies^[19–21]^ provided data for DFS with a cutoff *>*40% for determining PCNA expression. The result showed that PCNA expression had no correlation with DFS of osteosarcoma (RR = 1.00, 95% CI 0.72–1.39; *P* = .998). No evidence of statistically significant heterogeneity (*I*^2^ = 0.0%, *P* for heterogeneity = .733) was found between studies (Fig. 4).

#### 3.5.5 Cutoff >50% in OS

In total, 4 studies^[24,25,27,29]^ reported data for OS with a cutoff *>*50% for determining PCNA expression. Compared with low PCNA expression, high PCNA expression was correlated with a poor prognosis in OS of osteosarcoma (RR = 1.84, 95% CI 1.41–2.40; *P* = .000). No evidence of statistically significant heterogeneity (*I*^2^ = 0.0%, *P* for heterogeneity = .947) was found between studies (Fig. 4).

#### 3.5.6 Cutoff >75% in OS

Three studies^[23–25]^ provided valuable information for OS with cutoff >75% for determination of PCNA expression. Meta-analysis of these 3 studies showed no statistical association between PCNA expression and OS of osteosarcoma (RR = 1.68, 95% CI 1.16–2.43; *P* = .006). No evidence of statistically significant heterogeneity (*I*^2^ = 14.5%, *P* for heterogeneity = .311) was found between studies (Fig. 4).

### 3.6 Sensitivity analyses and publication bias

**Figure 5:**
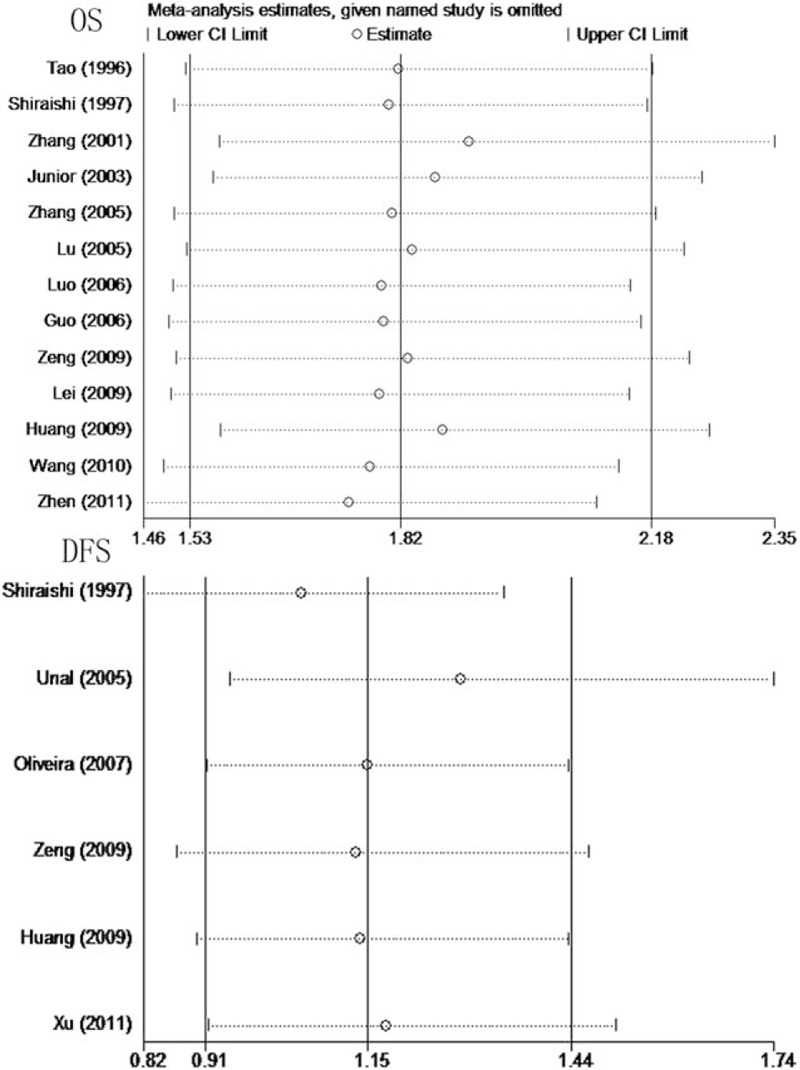
Results of sensitivity analyses for OS and DFS in osteosarcoma. DFS = disease-free survival, OS = overall survival.

**Figure 6:**
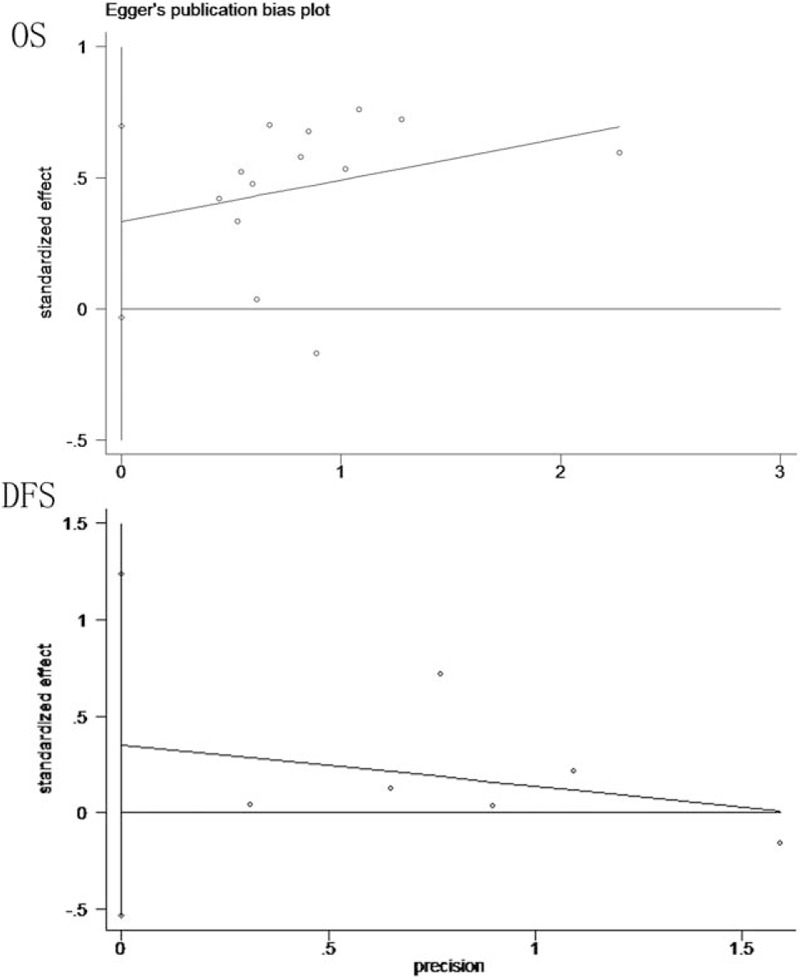
Egger publication bias plot for OS and DFS in osteosarcoma. DFS = disease-free survival, OS = overall survival.

Sensitivity analyses showed no statistical significance with the result, remaining highly consistent when each study was removed one by one (Fig. 5). Publication bias evaluated by Egger test showed no statistical significance (*P* = .075 and *P* = .330, respectively) (Fig. 6).

## 4 Discussion

Osteosarcoma, often occurring in the pediatric age group, is the most common primary malignant bone tumor, and is the second leading cause of cancer-related death in teenagers.^[33,34]^ With improvement of treatment with numerous methods, including surgical resection of primary and tumors distant metastases, radiotherapy, and chemotherapy, the OS rate of osteosarcoma has been significantly improved in the last decade, however, the prognosis for osteosarcoma patients is still poor with low OS. Currently, the ability to predict osteosarcoma prognosis is limited. Identifying the level of malignancy in osteosarcoma is of great importance for clinical decision making. It is important to discover new and reliable markers to predict the prognosis of osteosarcoma. Therefore, increasing attention has been given to explore better prognostic markers and find the best therapeutic targets to improve the accuracy of first diagnosis and identify the prognosis of osteosarcoma.

Multiple studies have attempted to explore the relationship between PCNA expression and OS or DFS in osteosarcoma but the sample size of these studies was small, which weakened the reliability of their result. Currently, the issue remains controversial and whether the result is consistent in subgroup analyses is not known. Systematic review and meta-analysis is a quantitative statistical approach that collects all possible traits of similar studies to resolve controversial issues with appropriate quantitative measures of the average effect, and has been widely used to evaluate multiple biomarkers.^[22,23]^ To the best of our knowledge, no meta-analysis has been performed to resolve this unanswered question by pooling the available data of relevant studies. Therefore, a meta-analysis including high quality studies was needed to comprehensively evaluate the association of PCNA expression and prognosis of osteosarcoma patients. Thus, to assess this association more precisely, we comprehensively researched all relevant published studies to conduct a meta-analysis.

Tumor antigens play an important role in tumor occurrence, development, and metastasis and among these, PCNA is one of the most important. Mathews et al^[12]^ recognized the PCNA and Waseem et al^[35]^ isolated and identified 11 kinds of anti-PCNA antibodies, of which PC-10 (Mo-Ab) is the most widely used. PCNA is a cell cycle regulatory protein, whose expression shows a periodic change with the DNA replication phase, which plays an important role in the cell transition from the G1 to S phase. As a co-factor for DNA polymerase, PCNA regulates the synthesis of the leading and lagging strands, which is essential for DNA duplication.^[36,37]^ Welkoborsky et al^[38]^ reported that PCNA expression is closely tied to the tumor prognosis, which can reflect the biological characteristics of the tumor cells. Based on a study of 49 osteosarcoma cases, Lopes suggested^[18]^ that PCNA expression was significantly associated with clinical stage, histological grade, and poor prognosis of osteosarcoma, which could evaluate tumor cell proliferation, and predict its biological behavior and prognosis.

In our investigation, 16 studies with 691 patients were selected for a meta-analysis. Meta-analysis of 13 studies showed that patients with high PCNA expression had a lower OS than those with low PCNA expression (RR = 1.82, 95% CI 1.53–2.18, *P* = .000).

In 1997, Shiraishi et al^[17]^ first performed immunohistochemical staining of osteosarcoma specimens with an anti-PCNA monoclonal antibody to describe utility of the PCNA labeling index for predicting prognosis in osteosarcoma.

The PCNA labeling index, p53 expression and p53 labeling index in immunohistochemical stained specimens were simple and feasible indicators of prognosis in osteosarcoma. In our study, 6 studies provided valuable information to estimate the prognostic role of PCNA expression in patients with osteosarcoma at disease-free survival (DFS). Meta-analysis of these 6 studies showed no statistical association between PCNA expression and DFS of osteosarcoma (RR = 1.15, 95% CI 0.91–1.44, *P* = .234). No obvious between-study heterogeneity was observed for OS and DFS (*I*^2^ = 42.5%, *P* for heterogeneity = .052; *I*^2^ = 36.4%, *P* for heterogeneity = .164, respectively). Sensitivity analysis demonstrated that the results were stable and did not change upon omitting each study. Subgroup analyses based on different follow-up and cutoffs to determine PCNA positivity were performed, and the results were stable and did not change. This meta-analysis demonstrated that osteosarcoma patients with cutoff >10, 25, 50, 75 of PCNA expression were significantly associated with low OS rate. No significant association of DFS and osteosarcoma patients with cutoffs >5 and 40 of PCNA expression was found which is consistent with the previous conclusion. These findings from our meta-analysis facilitated precise assessment of the value of PCNA expression in patients with osteosarcoma.

Several limitations exist in this meta-analysis. First, few studies were included and their sample sizes were also small. Some subgroup analyses with only 2 studies in the meta-analysis may make conclusions less reliable. Second, many studies were excluded because they did not provide enough data to calculate the RR and 95% CI, which might introduce biases in the conclusion. Third, even though we tried to retrieve the data not provided in the original articles from the investigators, some data such as gender, inclusion period and Enneking stage were not accessible. Finally, there was variability in the antibodies used against PCNA and their sources; different studies used antibodies from different companies. Moreover, the stages of osteosarcoma and their follow-up also varied in these investigations. Therefore, heterogeneity was unavoidable.

## 5 Conclusion

In summary, the findings from this meta-analysis suggested that PCNA expression is an effective biomarker for poor prognosis in patients with osteosarcoma for OS. However, more large-scale studies are needed to further support this conclusion.
